# *Gingko biloba*-inspired lactone prevents osteoarthritis by activating the AMPK-SIRT1 signaling pathway

**DOI:** 10.1186/s13075-022-02890-y

**Published:** 2022-08-18

**Authors:** Zhijian Zhao, Yang Liu, Yingjie Lu, Mingzhuang Hou, Xu Shen, Huilin Yang, Qin Shi, Yijian Zhang, Fan He, Xuesong Zhu

**Affiliations:** 1 Department of Orthopaedics, The First Affiliated Hospital of Soochow University, Soochow University, Suzhou, 215006 China; 2 Orthopaedic Institute, Medical College, Soochow University, Suzhou, 215007 China; 3 Department of Orthopaedics, Suzhou Dushu Lake Hospital, Dushu Lake Hospital Affiliated to Soochow University, Suzhou, China

**Keywords:** Bilobalide, Osteoarthritis, Extracellular matrix, AMPK, SIRT1

## Abstract

**Background:**

Uncoupled extracellular matrix (ECM) causes cartilage degeneration and osteoarthritis (OA) by suppressing the synthesis and activating the degradation of ECM components. *Gingko biloba* is a natural Chinese herb with a variety of biological functions; however, the extent to which it can protect against OA and the mechanisms involved are unknown.

**Methods:**

In our study, using bioinformatics tools, we were able to identify an important lactone, bilobalide (BB), from *Gingko biloba*. In vitro experiments were performed to evaluate the potential therapeutic effects of BB on ECM homeostasis. In vivo experiments were conducted to assess the protection of systemic administration of BB on cartilage degeneration. Molecular mechanisms underlying BB-regulated anti-arthritic role were further explored.

**Results:**

In interleukin-1β-incubated human chondrocytes, in vitro treatment with BB increased the expression of cartilage anabolic proteins, while inhibiting the activities of ECM degrading enzymes. In a mice model, systemic administration of BB, in vivo, prevented post-traumatic cartilage erosion and attenuated the formation of abnormal osteophytes in the subchondral bone. Mechanistically, the activation of the adenosine 5′-monophosphate-activated protein kinase (AMPK)-sirtuin 1 (SIRT1) signaling pathway was involved in the anti-arthritic effects of BB. In vitro, blocking BB’s chondroprotection with the AMPK-specific inhibitor Compound C abrogated it.

**Conclusions:**

These results demonstrated that BB extracted from *Gingko biloba* regulates ECM balance to prevent OA by activating the AMPK-SIRT1 signaling pathway. This study proposed the monomer BB, a traditional Chinese medicine, as a *de novo* therapeutic insight for OA.

**Graphical Abstract:**

Schematic representation of the experimental design. Based on the bioinformatic analysis, bilobalide (BB), a natural herb *Gingko biloba*-derived ingredient, was identified as a candidate for treating osteoarthritis. In vitro, BB treatment not only facilitates cartilage extracellular matrix synthesis but also inhibits proteolytic enzyme activities. In vivo intraperitoneal injection of BB improves cartilage degeneration and subchondral bone sclerosis. BB, in particular, had anti-arthritic effects by activating the AMPK-SIRT1 signaling pathway.
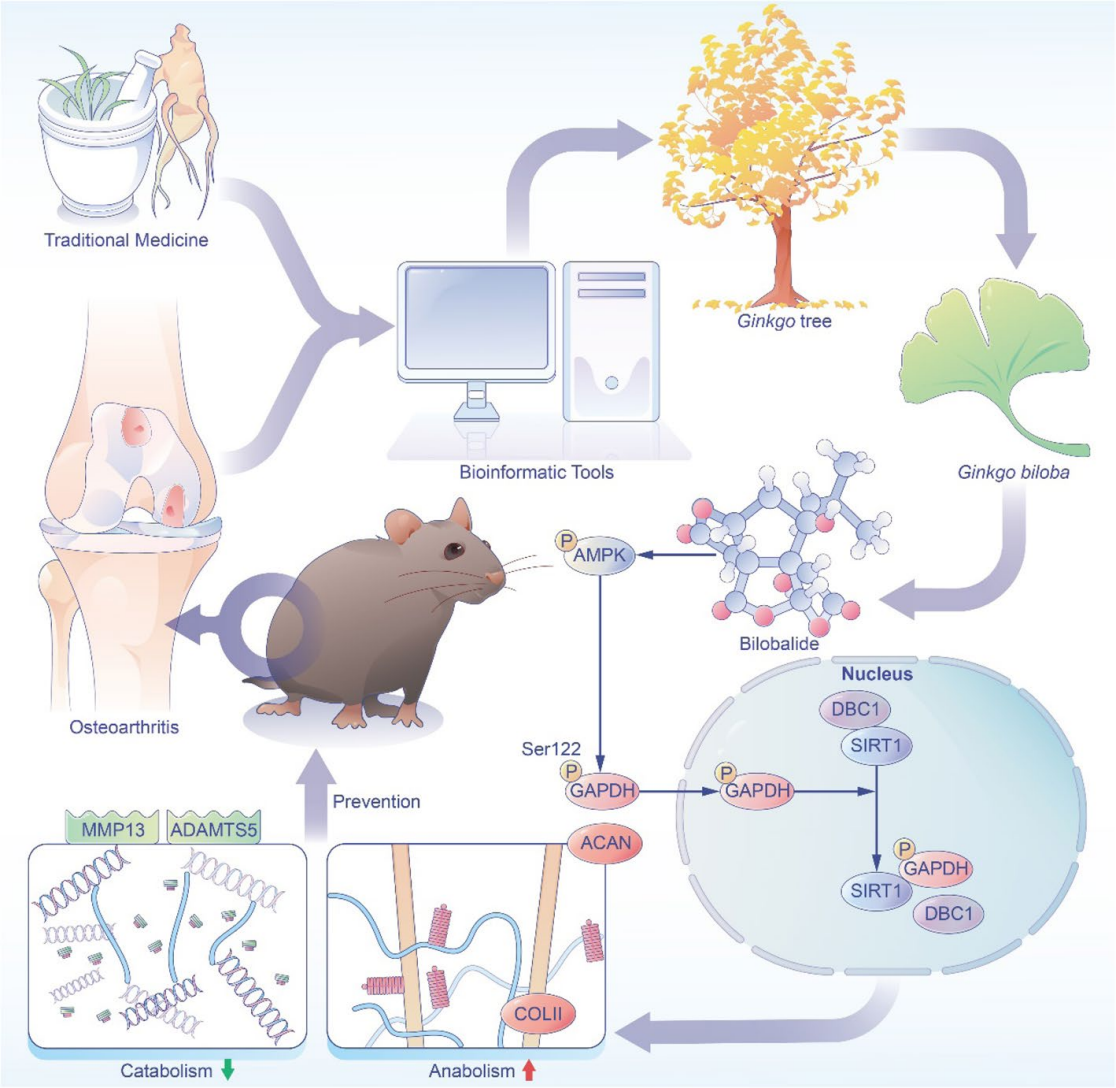

**Supplementary Information:**

The online version contains supplementary material available at 10.1186/s13075-022-02890-y.

## Introduction

Osteoarthritis (OA) is a significant and intractable disease caused by a variety of factors such as aging, obesity, trauma, and excessive exercise. Statistically, knee OA has doubled in prevalence in the last half-century [1]. Increased incidence of OA is expected to place a significant burden on society as life expectancy rises. Pain, stiffness, and functional impairment of the affected joint plagued patients with OA [2]. Unfortunately, the treatment options for OA have been restricted to oral analgesics or joint replacement surgery so far [3]. The primary feature of OA is the disruption of the cartilage extracellular matrix (ECM), which manifests as collagenase-mediated broken collagen fibers and aggrecanase-induced glycosaminoglycan degradation [4]. At the same time, abnormal osteophytes of the subchondral bone and the presence of synovial inflammation synergistically accelerate the progression of OA [5]. Thus, exploring new drugs or molecules to antagonize OA is on the horizon.

Traditional Chinese medicine (TCM) has been practiced for over 2000 years in ancient China and has been shown to have curative therapeutic effects [6]. Recently, the establishment of the TCM integrated database (TCMID) and TCM systems pharmacology (TCMSP) has aided research into herb molecular mechanisms [7]. OA has been treated with a variety of TCM formulas, such as *Corni Fructus* [8], *Cortex Daphnes* [9], and *Safflower* [10], according to the kidney-deficiency syndrome in TCM theory. *Ginkgo* (*Ginkgo biloba*) is an ancient living tree whose fan-shaped leaves are used to extract bioactive compounds such as flavonoids and terpenoids [11]. Bilobalide (BB), a lactone extracted from *Ginkgo biloba* leaves, has potent anti-inflammatory and antioxidant properties [12]. BB is used to treat neurological diseases such as Alzheimer’s disease [13] and multiple sclerosis [14] by reducing neuronal inflammation and damage. Moreover, BB protects chondrogenic (ATDC5) cell lines from proinflammatory interleukin (IL)-17-induced inflammatory injury; however, the protective effects of BB on cartilage metabolism and the underlying mechanisms are unknown.

Chondrocyte self-renewal and ECM synthesis are both driven by energy metabolism. Adenosine 5′-monophosphate-activated protein kinase (AMPK) is the master regulator of energy metabolism, maintaining the balance between AMP and adenosine triphosphate to coordinate energy usage and ECM homeostasis [15]. AMPK is a heterotrimeric complex composed of a catalytic subunit (α subunit) and regulatory subunits (β and γ subunits), of which AMPKα are ubiquitously expressed in articular cartilage and influences the progression of OA. In surgically or aging-induced OA models, mice with cartilage-specific knockout of AMPKα (*AMPKα*^−/−^) showed accelerated severity [16]. Through AMPK-dependent energy shifting from glycolysis to mitochondrial respiration [17], overexpression of 4-methylumbelliferone or hyaluronan synthase-2 prevents IL-1β-induced ECM degradation in chondrocytes. Similarly, metformin inhibits post-traumatic OA progression in mice and non-human primates by activating the AMPK signaling [18]. Sirtuin 1 (SIRT1), an NAD^+^ dependent histone deacetylase, acts as a critical signaling molecule downstream of AMPK. Deletion of SIRT1 in cartilage (*SIRT1*^−/−^) aggregates the development of OA in mice under mechanical stress and as they age [19]. In a recent study from our laboratory, it was discovered that synovium stem cell-derived ECM-enhanced anti-inflammatory properties in chondrocytes are dependent on the SIRT1 pathway [20]. Recent literature has reported that BB has a positive effect on AMPK activity [21], but the exact mechanisms and the role of SIRT1 remain unknown.

This study aimed to identify and investigate the potential therapeutic effects of *Gingko*-derived lactone on OA. BB was recognized as the candidate molecule using bioinformatics analysis tools. BB’s chondroprotective effects were evaluated in both an IL-1β and a post-traumatic OA model. The key pathways, AMPK-SIRT1, were screened using whole-genome sequencing. Finally, the mechanisms by which BB regulates ECM equilibrium and prevents the progression of OA were confirmed by specific AMPK inhibition with Compound C.

## Material and methods

### Bioinformatic analysis

The TCMID, TCMSP, Genecard database, and online Mendelian inheritance in man tools were used to find potential targets. With the help of the Cytoscape software (version 3.7.2), a medicine-ingredients-targets-disease (MITD) interaction map was created. The search tool for the retrieval of interacting genes/proteins database was used to gather protein-protein interaction networks. The R package (version 4.1.1) was used to perform the gene ontology (GO) and Kyoto encyclopedia of genes and genome (KEGG) analysis.

### Cell isolation and culture

The study protocol was approved by the ethics committee of The First Affiliated Hospital of Soochow University. Human cartilage samples were carefully cut into fragments of 1 mm^3^ from tibial plateaus of six patients (four females and two males) undergoing knee replacement surgery. Cartilage fragments were washed in phosphate-buffered saline (PBS) for 5 min and digested with 0.4% collagenase II (Gibco, Invitrogen) for 12 h at 37°C. The released cells were filtered through a 100-μm nylon mesh (BD, Biosciences, San Jose, CA, USA). Cells were seeded into 75-cm^2^ culture flasks (CoStar, Tewksbury, MA, USA) and cultured in Dulbecco’s modified Eagle’s medium/nutrient mixture F-12 with 10% fetal bovine serum (Gibco, heat-inactivated), penicillin (100 U/mL), and streptomycin (100 U/mL) at 37°C in a 5% carbon dioxide atmosphere. Primary cells were trypsinized with 0.25% trypsin-ethylenediamine tetraacetic acid (EDTA, Invitrogen) followed by centrifugation. For the following experiments, chondrocytes at passage one were used.

### Cell treatment

Chondrocytes were stimulated with 10 ng/mL recombinant human IL-1β (Peprotech, Rocky Hill, NJ, USA) to create an in vitro arthritic environment. At a stock concentration of 30 mM, BB (Sigma-Aldrich) was dissolved in dimethyl sulfoxide (DMSO). BB was applied to cells in a gradient concentration of 10 μM, 20 μM, and 40 μM. Chondrocytes were given the AMPK-specific inhibitor Compound C (10 μM, Sigma-Aldrich, P5499) to block the AMPK signaling pathway.

### Cell proliferation

To detect cell proliferation, the Cell Counting Kit-8 (CCK-8, Beyotime, Shanghai, China) was used. Chondrocytes were seeded into a 96-well plate. The next day, cells were stimulated with IL-1β or BB at various concentrations for seven days. The cells were incubated in a 10% CCK-8 solution for 2 h at 37°C on days 1, 3, 5, and 7. A microplate spectrophotometer (Bio Tek, Winooski, VT, USA) was used to measure the absorbance at 450 nm.

### Immunofluorescence staining

Chondrocytes were fixed with 4% paraformaldehyde for 30 min, then permeabilized with 0.1% Triton X-100 (Sigma-Aldrich) and blocked with bovine serum albumin for 1 h at room temperature. Cells were then incubated with diluted primary antibodies: anti-COLII (ab34712, Abcam, 1:500), anti-MMP13 (ab21962, 1:500), anti-P-AMPKα (ab23875, 1:500), and anti-SIRT1 (ab110304, 1:500) for 2 h. The cells were then washed with PBS before being incubated with FITC-phalloidine (RM02836, ABclonal, 1:500) and a secondary antibody (ab150075, 1:500) for 2 h. Nuclei were counterstained with 4′,6-diamidino-2-phenylindole (Thermo Fisher Scientific) for 1 min. A Zeiss Axiovert 40CFL microscope (Zeiss, Oberkochen, Germany) was used to capture fluorescent images.

### SIRT1 activity measurement

Chondrocytes were washed with PBS and the proteins were extracted with a nuclear extraction kit. Protein concentrations were determined using a BCA kit (Beyotime). SIRT1 activity was measured with a SIRT1 fluorimetric activity assay (JM5979, Yuduobio, Shanghai, China). The absorbance was measured at 405 nm using a microplate reader.

### Quantitative real-time reverse transcription-polymerase chain reaction (RT-PCR)

Total RNA was extracted from cells using the Trizol reagent (Invitrogen) according to the standard protocol. RevertAid First Strand complementary DNA Synthesis Kit (Thermo Fisher Scientific) was used to obtain the first strand of cDNA. RT-PCR was performed with Advanced Universal SYBR Green Supermix (Bio-Rad) on a CFX96 quantitative-PCR machine (Bio-Rad). The Ct (2 ^−ΔΔCt^) method was used to calculate all gene expression levels after normalizing them with *GAPDH* as a standard control. Supplementary Table 1 contains the primer sequences.

### Western blot

Radioimmunoprecipitation assay buffer was used to extract total proteins from chondrocytes (Beyotime). A BCA Protein Kit was used to determine protein concentrations (Beyotime). A 10% sodium dodecyl sulfate-polyacrylamide gel electrophoresis was used to separate equivalent amounts of protein samples, which were then transferred onto nitrocellulose membranes (Bio-Rad). Membranes were incubated in blocking buffer (Beyotime) for 1 h and probed with several diluted primary antibodies overnight at 4°C, including anti-COLII (ab188570, 1:5000), anti-ACAN (ab3778, 1:3000), anti-MMP13 (ab39012, 1:5000), anti-ADAMTS5 (ab41037, 1:5000), anti-P-AMPKα (phosphorylated T183 + T172) (ab23875, 1:5000), anti-AMPKα (ab131512, 1:5000), and anti-α-Tubulin (ab7291, 1:5000). After washing with phosphate-buffered saline with Tween 20 (Beyotime), the blots were incubated with horseradish peroxidase-conjugated secondary antibody for 1 h. Enhanced chemiluminescence (SuperSignal West Pico Substrate, Thermo Fisher Scientific) was used to visualize protein bands. Image J software (National Institutes of Health) was used to analyze gray values, which were then normalized to the expression levels of α-Tubulin.

### RNA sequencing

The gene expressions of human chondrocytes were analyzed using Affymetrix Gene Chip microarrays (Affymetrix, Santa Clara, CA, USA) after they were treated with 40 μM BB or PBS. The Agilent Bioanalyzer 2100 (Agilent Technologies, Santa Clara, CA, USA) was used to measure RNA levels, which were then analyzed using the Significant Analysis of Microarray software. Gene expressions with a fold change (>2) were considered valid. Targetscan (www.Targetscan.org) was used to predict the target genes with differential expression, which were obtained from the Gene Expression Omnibus database (www.ncbi.nlm.nih.gov/gds/). The RNA sequencing was performed with the assistance of the Wekemo Tech Group Co., Ltd. (Shenzhen China).

### Animal model

All animal procedures were in accordance with applicable regulations and were approved by the ethics committee of Soochow University. Adult male C57BL/6J mice (seven weeks old) were purchased from the Experimental Animal Center of Soochow University. Destabilization of medial meniscus (DMM) surgery was used on the animals to induce post-traumatic OA, according to previous procedures [22]. Animals were anesthetized with 2.0% isoflurane on a nitro-oxygen/oxygen mixture using an inhalation anesthesia machine (RWD Life Science, Shenzhen, China). For the DMM group, the medial meniscotibial ligament (MML) was transected with a micro scissor. The Sham group underwent the same procedures except for the retention of MML. All the surgeries were performed by the same surgeon, who was blinded to the experimental grouping.

### Administration of BB

To make a stock solution (10 mg/mL), BB was dissolved in DMSO (Sigma-Aldrich). After surgery, mice were randomly assigned to one of the four groups: Sham group, Sham + BB group, DMM group, or DMM + BB group. The stock solution was diluted to the desired concentration using 0.9% saline. For 8 weeks, mice were given an intraperitoneal injection of BB (5 mg/kg) or an equivalent amount of saline twice a week. Animals were sacrificed at week eight, and the knee joint samples were collected for subsequent analysis.

### Micro-computed tomography (CT) scan

The Skyscan-1176 scanning system (Kontich, Belgium) was used to scan knee joint samples at 50 kV/200 μA and 9 μm resolution. CTAN v1.13.8.1 software was used to analyze scanning data quantitatively and Mimics 16.0 software was used to reconstruct it. The medial tibial plateau was selected as the region of interest for the reconstruction of the three-dimensional model.

### Histological staining

Samples of the knee joint were fixed in 10% formalin for 48 h before being decalcified in 10% EDTA (Sigma-Aldrich) for four weeks. Samples of paraffin-embedded knee joints were sectioned into slices of 6 μm thickness. Following the manufacturer’s procedures, the specimens were stained with hematoxylin and eosin, Safranin O (SO)/Fast Green (Sigma-Aldrich), and Toluidine Blue O (TB) after deparaffinization. An upright microscope (Zeiss Axiovert 200, Oberkochen, Germany) was used to obtain the experimental images. The cartilage degeneration degrees were assessed using the Osteoarthritis Research Society International (OARSI) scoring system, the hyaline cartilage (HC) versus calcified cartilage (CC) ratio, or the summed score [23].

### Immunohistochemical staining

Slides were heated for 30 min at 65°C, then treated with 1% hydrogen peroxide for 15 min before being incubated with testicular hyaluronidase (Sigma-Aldrich) for 60 min at 37°C. Anti-COLII (ab34712, 1:250), anti-MMP13 (ab21962, 1:250), anti-P-AMPKα (ab23875, 1:200), and anti-SIRT1 (ab110304, 1:200) were used to incubate slides at 4°C overnight after blocking with 1.5% goat serum. The next day, the slides were incubated for 60 min with a secondary antibody (Vector Laboratories, Burlingame, CA, USA), then for 30 min with the ABC kit (Vector Laboratories). The slides were developed using a DAB substrate kit (Vector Laboratories). Image J software was used to examine the percentage of positive cells.

### Statistical analysis

All numerical data are presented as mean ± standard deviation and analyzed using Prism GraphPad 9.0 software. Unpaired two-sided Student’s *t*-test was calculated to compare the two groups. Using one-way analysis of variance and Tukey’s post hoc test, differences between multiple groups were determined. *P* < 0.05 was considered statistically significant.

## Results

### Bioinformatic analysis between Ginkgo biloba and OA

**Fig. 1:**
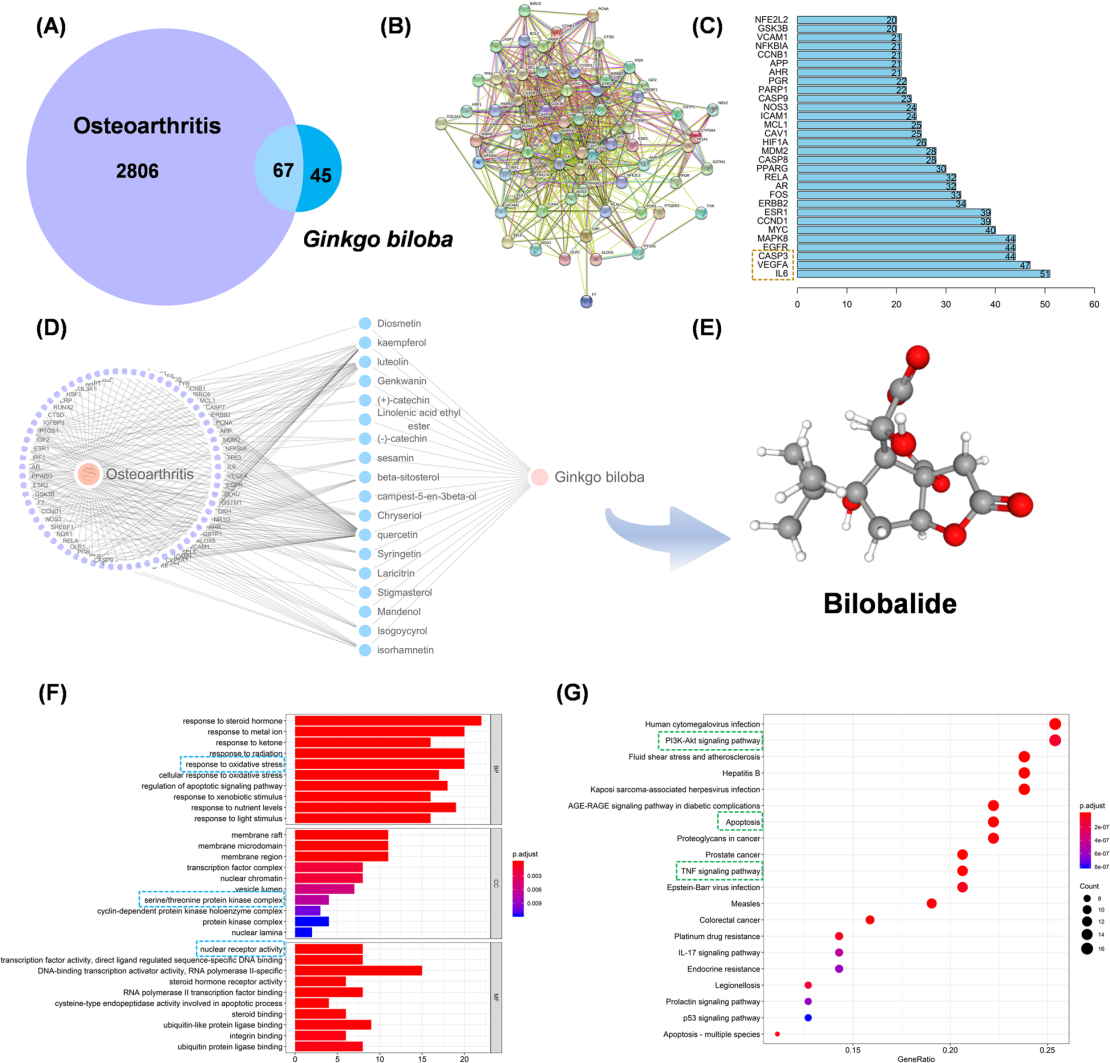
*Gingko biloba* and osteoarthritis (OA) were screened for potential ingredients and targets. **A** The intersection of the *Gingko biloba* and OA targets. **B** Protein-protein interaction map of the 67 candidates. **C** The number of nodes in which key proteins are ranked. **D** Medicine-Ingredient-Target-Disease model. **E** The structure of bilobalide. **F**, **G** Gene ontology and Kyoto encyclopedia of genes and genomics enrichment analysis according to the differentially expressed genes

A total of 67 intersected genes between *Ginkgo biloba* and OA were screened by drug target prediction (Fig. 1A). Protein interaction map identified 691 edges and 20.6 node degrees (Fig. 1B), in which IL-6, vascular endothelial growth factor A (VEGFA), and caspase 3 acted as the top three genes (Fig. 1C). There were 18 molecules and 67 targets in the MITD network (Fig. 1D). BB is a sesquiterpenoid with a molecular weight of 326.3 that is extracted from the *Ginkgo biloba* leaves (Fig. 1E). Response to oxidative stress (GO:0006979, biological process, BP), serine/threonine-protein kinase complex (GO:1902554, cellular component, CC), and nuclear receptor activity (GO:0004879, molecular function, MF) were found in GO enrichment analyses (Fig. 1F). According to KEGG enrichment, PI3K-AKT signaling pathway (hsa04151), apoptosis (hsa04210), and tumor necrosis factor (TNF) signaling pathway (hsa04668) may be involved in the interactions between *Ginkgo biloba* and OA (Fig. 1G).

### BB promoted anabolism and suppressed catabolism in IL-1β-stimulated chondrocytes

**Fig. 2:**
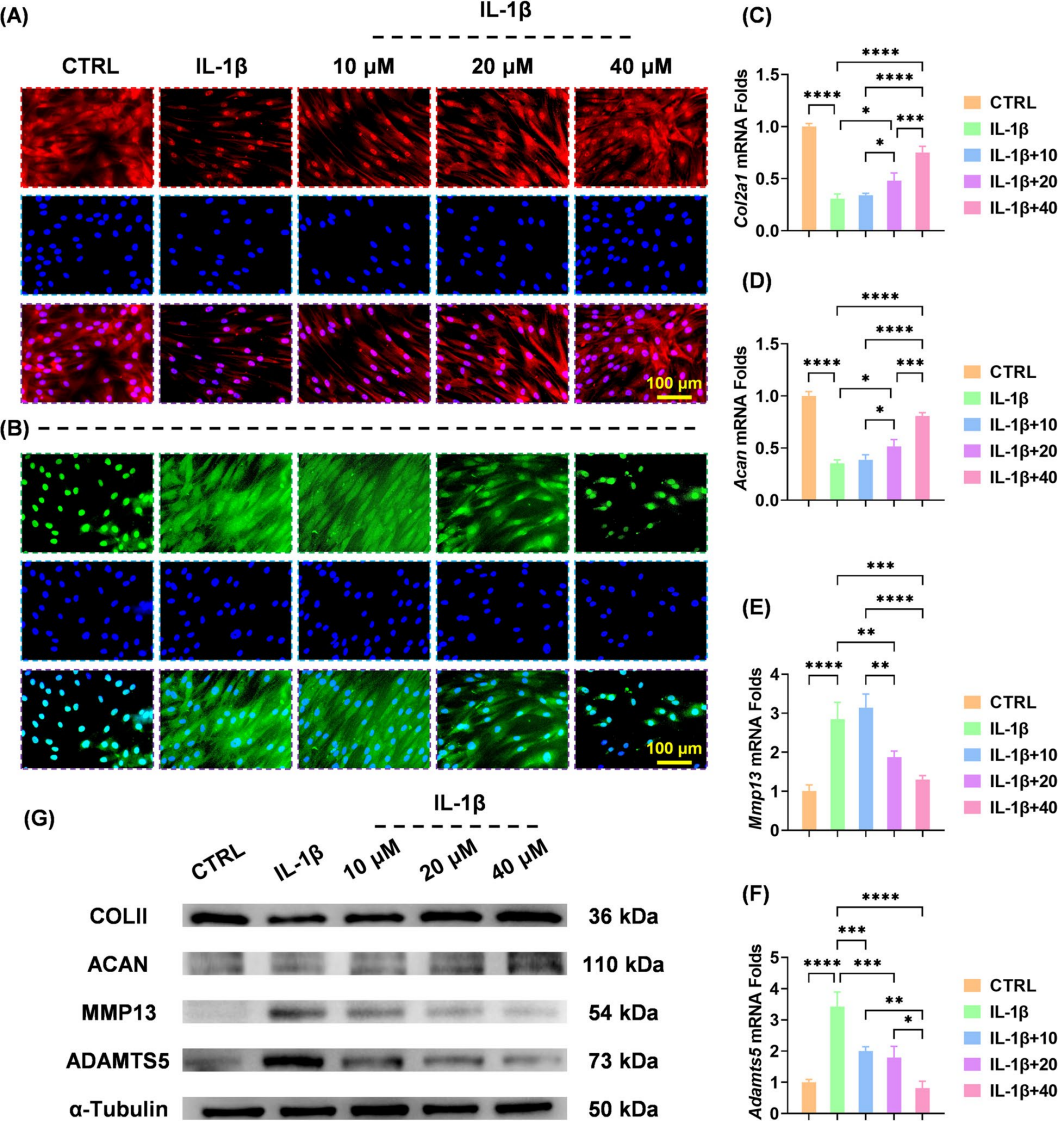
In an in vitro arthritic environment, bilobalide maintained the balance between anabolism and catabolism. **A**, **B** Immunofluorescence staining showed COLII or MMP13 expression in tandem. **C**–**F** Using real-time reverse transcription-polymerase chain reaction and GAPDH as an internal reference, the transcript levels of *Col2a1*, *Acan*, *Mmp13*, and *Adamts5* were quantified. **G** Western blot assays were used to determine the protein levels of COLII, ACAN, MMP13, and ADAMTS5. The values represent the mean ± standard deviation. Each in vitro experiment was repeated three times or more. Statistically, significant differences between the indicated groups are indicated by ^*^p < 0.05, ^**^p < 0.01, ^***^p < 0.001, or ^****^p < 0.0001

Human chondrocytes were treated with a gradient concentration of BB after being subjected to 10 ng/mL IL-1β. The CCK-8 assays showed that IL-1β-treated chondrocytes yielded a 28.1% higher cell proliferation at day five and a 49.6% higher cell proliferation at day seven than those in the CTRL group; however, BB did not affect cell proliferation at any time point (Fig. S1A-D). The IL-1β treatment disrupted the metabolic balance of cartilage ECM, as evidenced by decreased anabolic and increased catabolic activities. Intriguingly, the expression of COLII was upregulated by 23.4% at 10 μM, 63.5% at 20 μM, and 1.03-fold at 40 μM in BB-treated cells (Fig. 2A and S2A). BB, on the other hand, downregulated the expression of MMP13 in a dose-dependent manner (Fig. 2B and S2B). Moreover, 40 μM of BB upregulated the transcript levels of *Col2a1* by 1.44-fold (Fig. 2C) and *Acan* by 1.28-fold (Fig. 2D), respectively. The mRNA expressions of *Mmp13* (Fig. 2E) and *Adamts5* (Fig. 2F) were downregulated in BB-treated chondrocytes. Protein levels were found to be consistent with their mRNA expression using Western blot assays (Fig. 2G and S2C-F).

### In vivo application of BB delayed the progression of post-traumatic OA

**Fig. 3:**
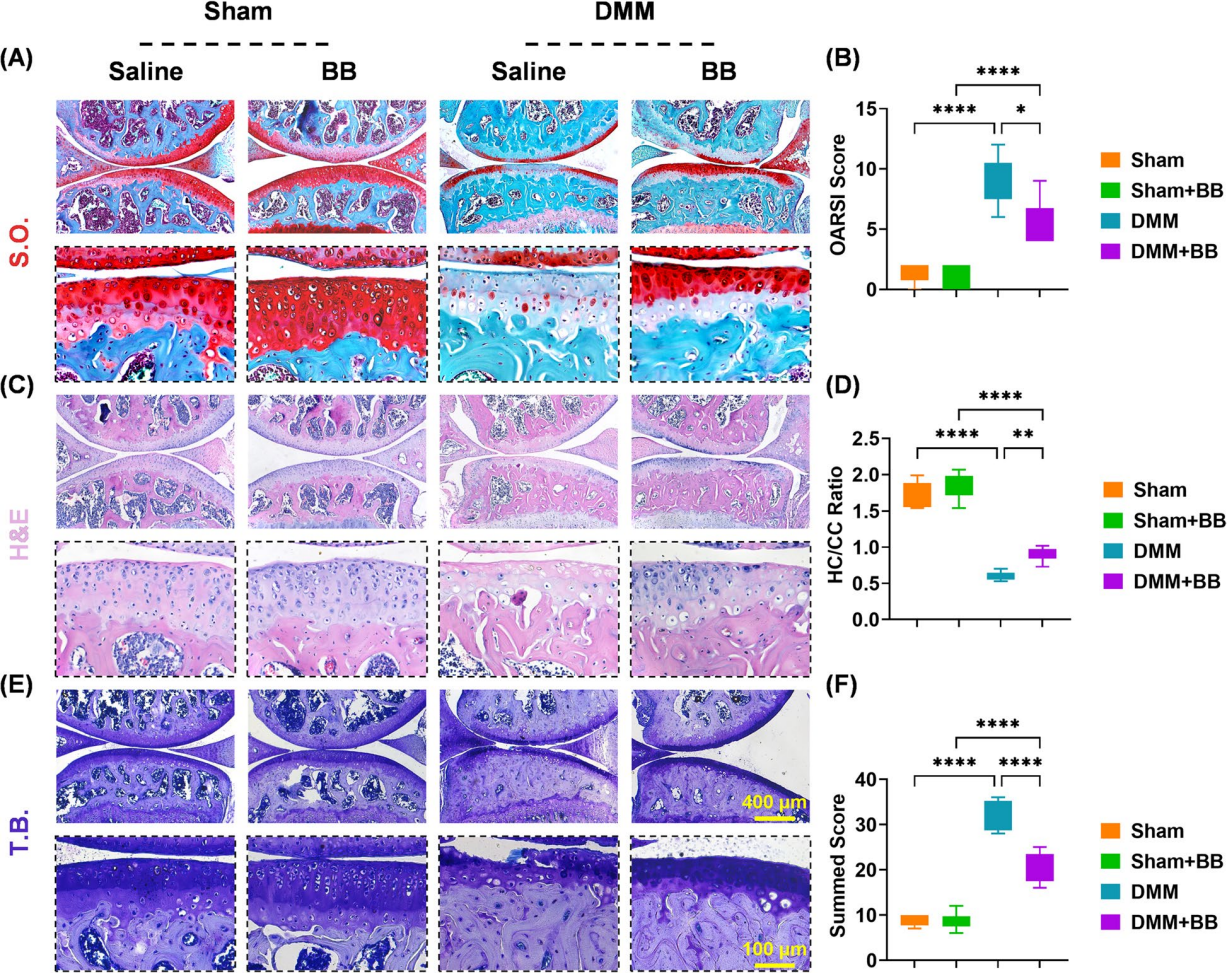
In post-traumatic mice models, bilobalide reduced cartilage erosion. **A**, **B** Representative images of articular cartilage stained with safranin O and fast green. The Osteoarthritis Research Society International score was quantified. **C**, **D** Representative images of articular cartilage stained with hematoxylin and eosin. The ratio of hyaline cartilage to calcified cartilage was measured. **E**, **F** Representative images of articular cartilage stained with toluidine blue. Quantification of the summed score. The values represent the mean ± standard deviation. Each in vivo experiment was repeated six times or more. Statistically, significant differences between the indicated groups are indicated by ^*^p < 0.05, ^**^p < 0.01, ^***^p < 0.001, or ^****^p < 0.0001

Sham or DMM-induced post-traumatic mice were intraperitoneally injected with 5 mg/kg BB or an equal volume of saline for 8 weeks to test the potential efficacy in preventing OA in vivo. When compared to the DMM group, the OARSI score was improved by 34.0% in the DMM + BB group (Fig. 3A, B). In comparison to the untreated mice, BB-injected mice showed a 50.1% higher HC versus CC ratio (Fig. 3C, D). Meanwhile, TB staining revealed that administration of BB upregulated the total score by 35.6% (Fig. 3E, F).

**Fig. 4:**
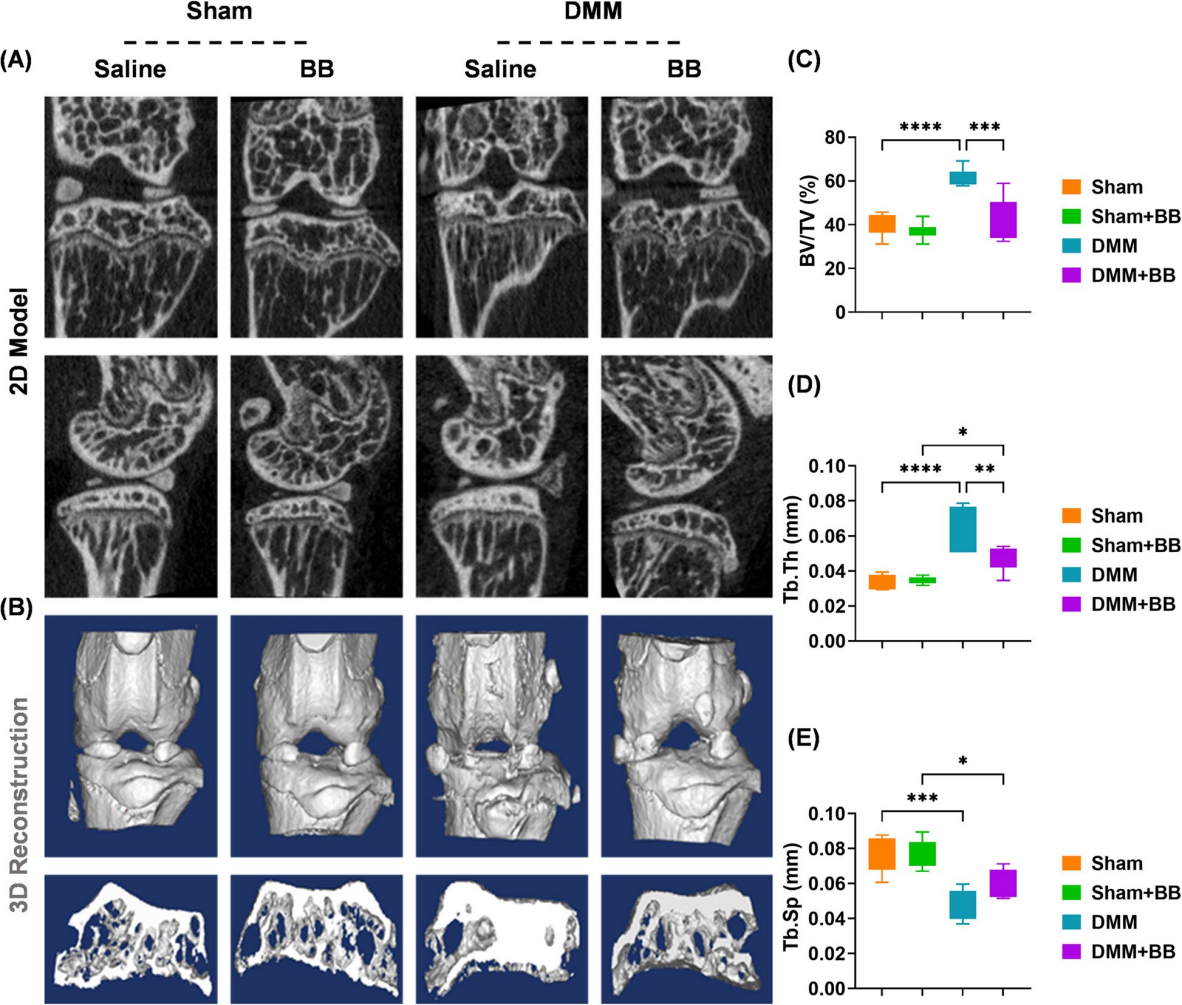
Bilobalide (BB) improved the destabilization of medial meniscus-induced subchondral bone sclerosis. **A** Two-dimensional images of the coronal and sagittal model. **B** Reconstructed three-dimensional model of the formation of osteocytes. **C**–**E** The impact of BB on bone volume ratio (%), trabecular thickness (mm), and trabecular separation (mm). The values represent the mean ± standard deviation. Each in vivo experiment was repeated six times or more. Statistically, significant differences between the indicated groups are indicated by by ^*^p < 0.05, ^**^p < 0.01, ^***^p < 0.001, or ^****^p < 0.0001

Subsequently, μCT scanning was performed to evaluate the alterations in the subchondral bone. Two-dimensional images in the sagittal and coronal planes showed that DMM-impaired subchondral bone sclerosis was alleviated by BB treatment (Fig. 4A). Three-dimensional reconstructed images confirmed that BB prevented the formation of osteophytes (Fig. 4B). The BV versus TV value was improved by 31.1%, the trabecular thickness (Tb. Th) value by 24.7%, and the trabecular separation (Tb. Sp) value by 24.2%, respectively, after treatment with BB (Fig. 4C–E).

### Whole transcriptome sequencing identified the involvement of AMPK-SIRT1

**Fig. 5:**
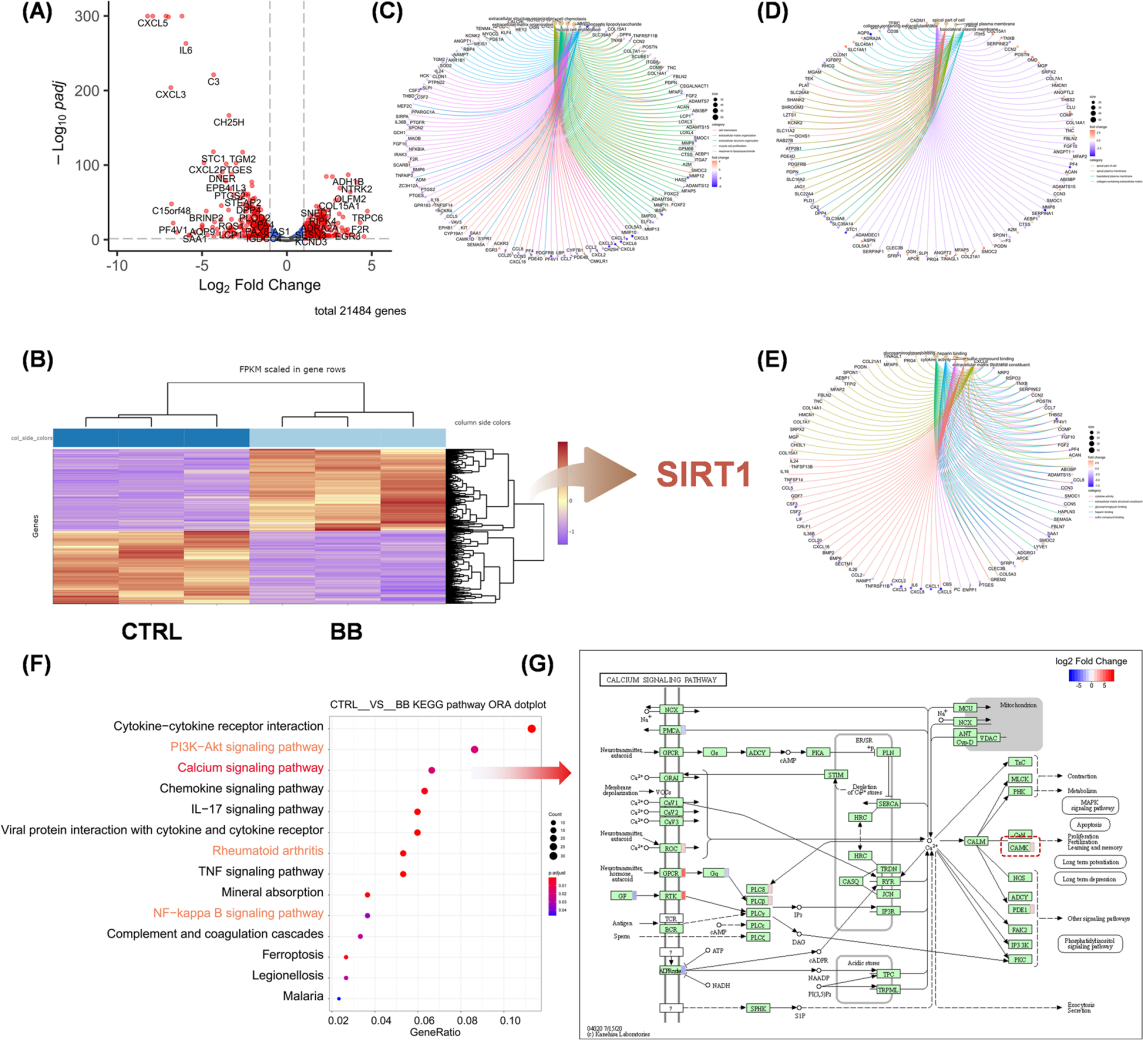
RNA sequencing of chondrocytes that had been subjected to bilobalide (BB). **A** The differentially expressed genes between the CTRL and BB groups. **C**–**E** Gene ontology enrichment analysis of the biological process, cellular component, and molecular function. **F** The differentially regulated pathways were discovered using the Kyoto encyclopedia of genes and genomics enrichment analysis. **G** The cAMP and cAMK proteins were found to be involved in the calcium signaling pathway

Transcriptome sequencing was performed to identify the key genes or pathways in BB-treated cells. After BB treatment, there were a total of 685 differentially expressed genes (DEGs), with 322 high-expressed genes and 363 down-expressed genes, as shown in the volcano plot (Fig. 5A) and heat map (Fig. 5B). Interestingly, the mRNA expression of SIRT1 was upregulated by 34.4% in BB-stimulated cells. ECM organization (GO:0030198, BP, Fig. 5C), collagen-containing ECM (GO:0062023, CC, Fig. 5D), and glycosaminoglycan binding (GO:0005539, MF, Fig. 5E) were among the several cartilage ECM-associated terms identified by the GO enrichment. Further KEGG pathway analysis indicated that PI3K-AKT, calcium signaling pathway, and rheumatoid arthritis (RA) all played a role in the chondroprotective effects of BB (Fig. 5F). According to a detailed analysis, energy-related components such as cyclic adenosine monophosphate (cAMP) and Ca^2+^/calmodulin-dependent kinase (cAMK) played an essential role in the calcium signaling pathway (Fig. 5G).

### BB-induced anti-arthritic effects depended on the activation of AMPK-SIRT1

**Fig. 6:**
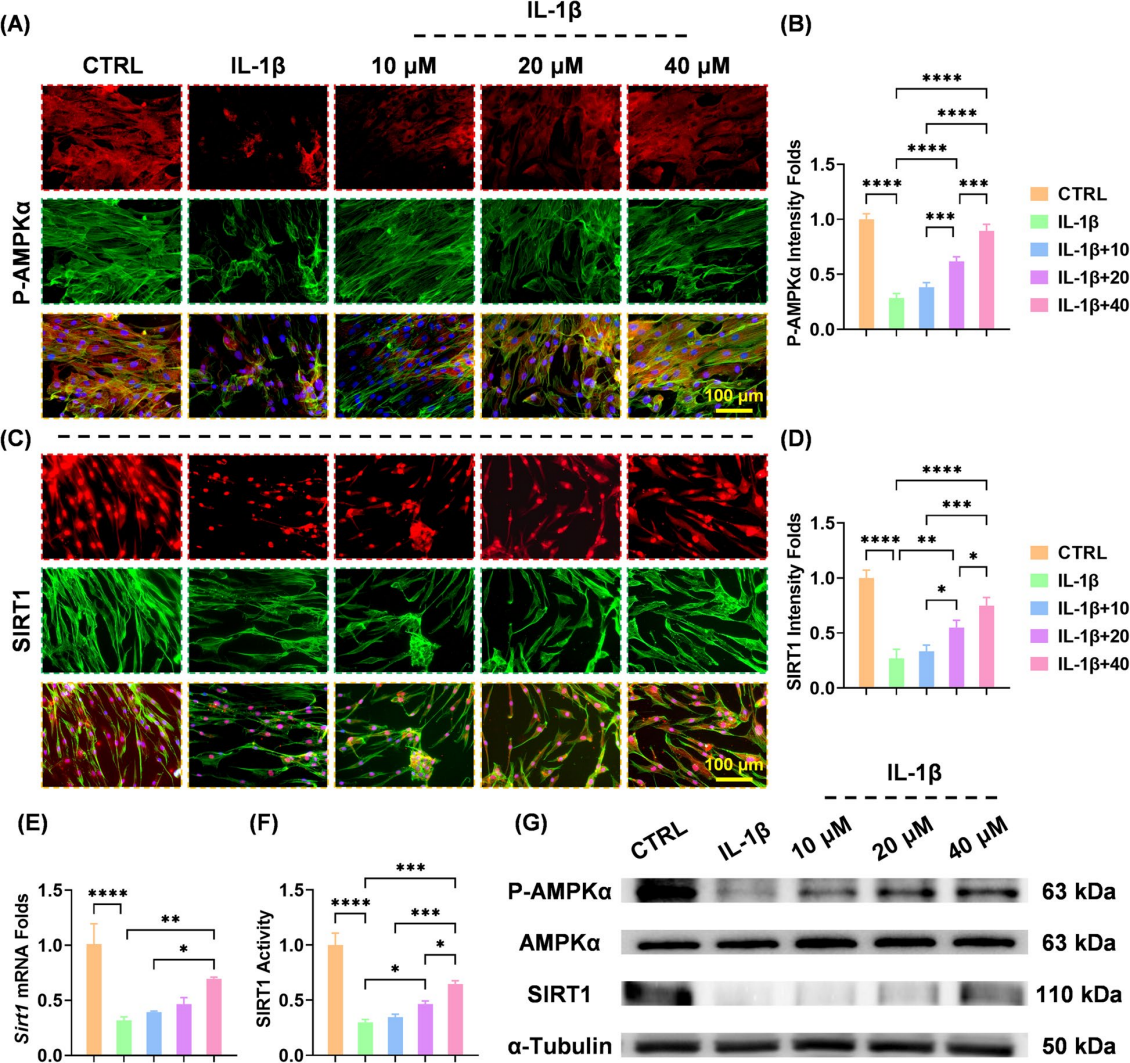
Bilobalide inhibited OA by activating the AMPK-SIRT1 pathway. **A**, **B** Immunofluorescence staining showed that bilobalide treatment increased the expression levels of phospho-AMPKα. **C**, **D** Immunofluorescence staining showed increased SIRT1 expression levels. **E** SIRT1 transcript levels were quantified using a real-time reverse transcription-polymerase chain reaction. **F** SIRT1 activity was quantified using a colorimetric measurement. **G** Western blot assays were used to determine the protein levels of P-AMPKα and SIRT1. The values represent the mean ± standard deviation. Each in vitro experiment was repeated three times or more. Statistically, significant differences between the indicated groups are indicated by ^*^p < 0.05, ^**^p < 0.01, ^***^p < 0.001, or ^****^p < 0.0001

The activity of phospho-AMPKα (P-AMPKα) and SIRT1 were measured to investigate the underlying mechanisms by which BB protected ECM homeostasis. Immunofluorescence staining showed that 40 μM of BB upregulated the expression of P-AMPKα by 2.15-fold (Fig. 6A, B). Meanwhile, BB increased SIRT1 expression level in chondrocytes exposed to IL-1β in a dose-dependent manner (Fig. 6C, D). RT-PCR assays indicated that chondrocytes treated with 40 μM of BB yielded a 1.18-fold higher SIRT1 mRNA level (Fig. 6E). The activity of SIRT1 was up-regulated by 1.16-fold after treatment with 40 μM BB (Fig. 6F). Western blot data confirmed that in vitro treatments with BB enhanced P-AMPKα and SIRT1 protein levels (Fig. 6G and S3A-B).

**Fig. 7:**
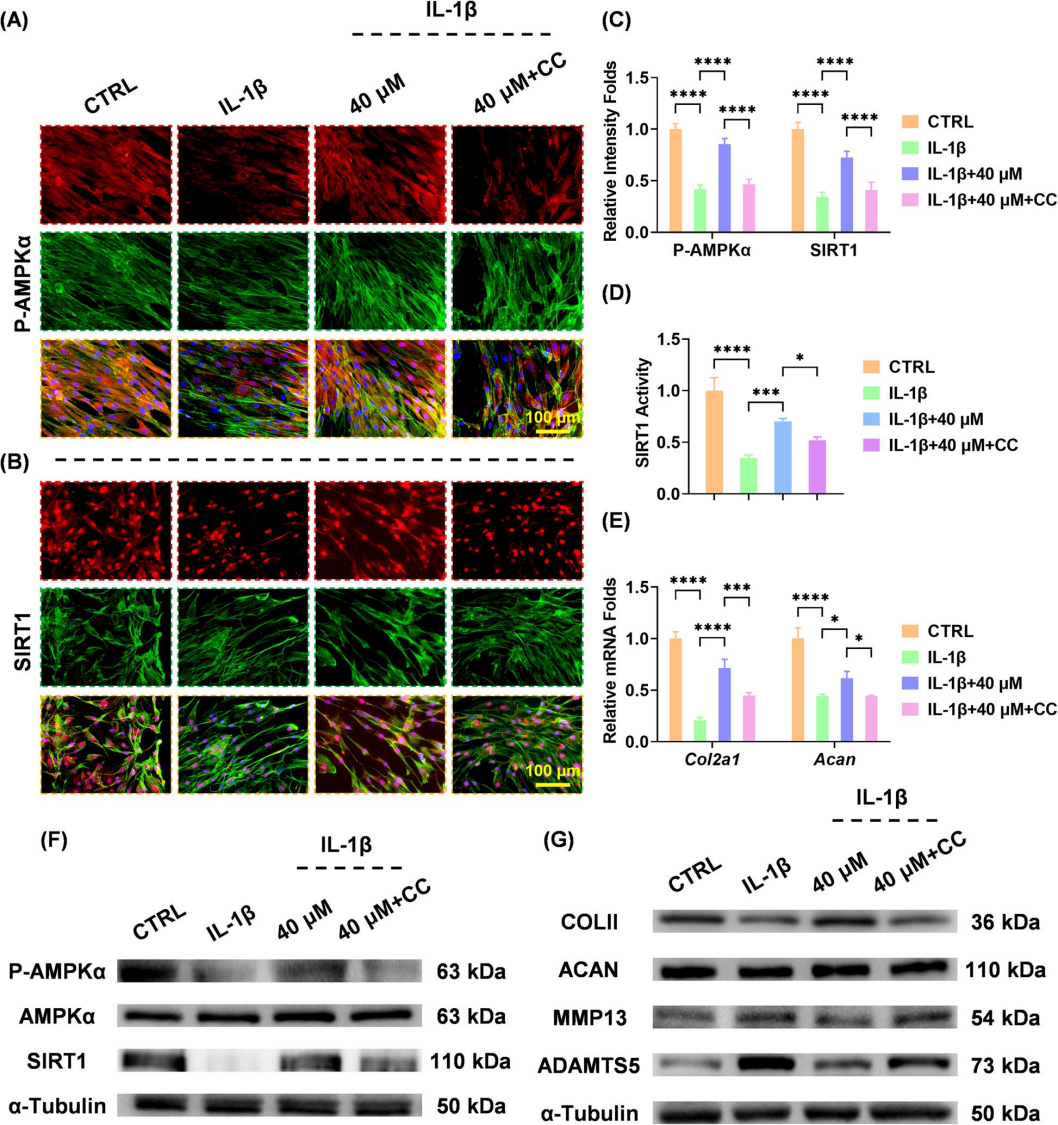
The anti-arthritic effects of bilobalide were abolished when AMPK was inhibited. **A** Immunofluorescence staining showed that Compound C treatment reduced phospho (P)-AMPKα expression levels. **B** Immunofluorescence staining showed SIRT1 expression levels that were inhibited. **C** Quantitative analysis of P-AMPKα and SIRT1 activity. **D** SIRT1 activity was inhibited by the treatment with Compound C. **E** Using real-time reverse-transcription-polymerase chain reaction and GAPDH as an internal reference, the transcript levels of *Col2a1* and *Acan* were determined. **F**, **G** Western blot assays were used to determine the protein levels of P-AMPKα, AMPKα, SIRT1, COLII, ACAN, MMP13, and ADAMTS5. The values represent the mean ± standard deviation. Each in vitro experiment was repeated three times or more. Statistically, significant differences between the indicated groups are indicated by ^*^p < 0.05, ^**^p < 0.01, ^***^p < 0.001, or ^****^p < 0.0001

Further experiments were designed to determine the role of the AMPK-SIRT1 signaling pathway. An AMPK-specific antagonist, compound C, was used for BB-treated chondrocytes. Compound C inhibited the activity of P-AMPKα and downstream SIRT1 (Fig. 7A–D). Moreover, inhibiting AMPK downregulated the expression of anabolic genes *Col2a1* by 37.6% and *Acan* by 28.0% (Fig. 7E), but upregulated the transcript levels of catabolic genes *Mmp13* and *Adamts5* (S4A-B). Western blot assay showed that the phosphorylation of AMPKα and SIRT1 were decreased by compound C (Fig. 7F and S4C-D) and the BB-induced ECM protection was lost as a result (Fig. 7G and S4E-H).

### BB regulated the cartilage ECM equilibrium by upregulating the AMPK-SIRT1 pathway in vivo

**Fig. 8:**
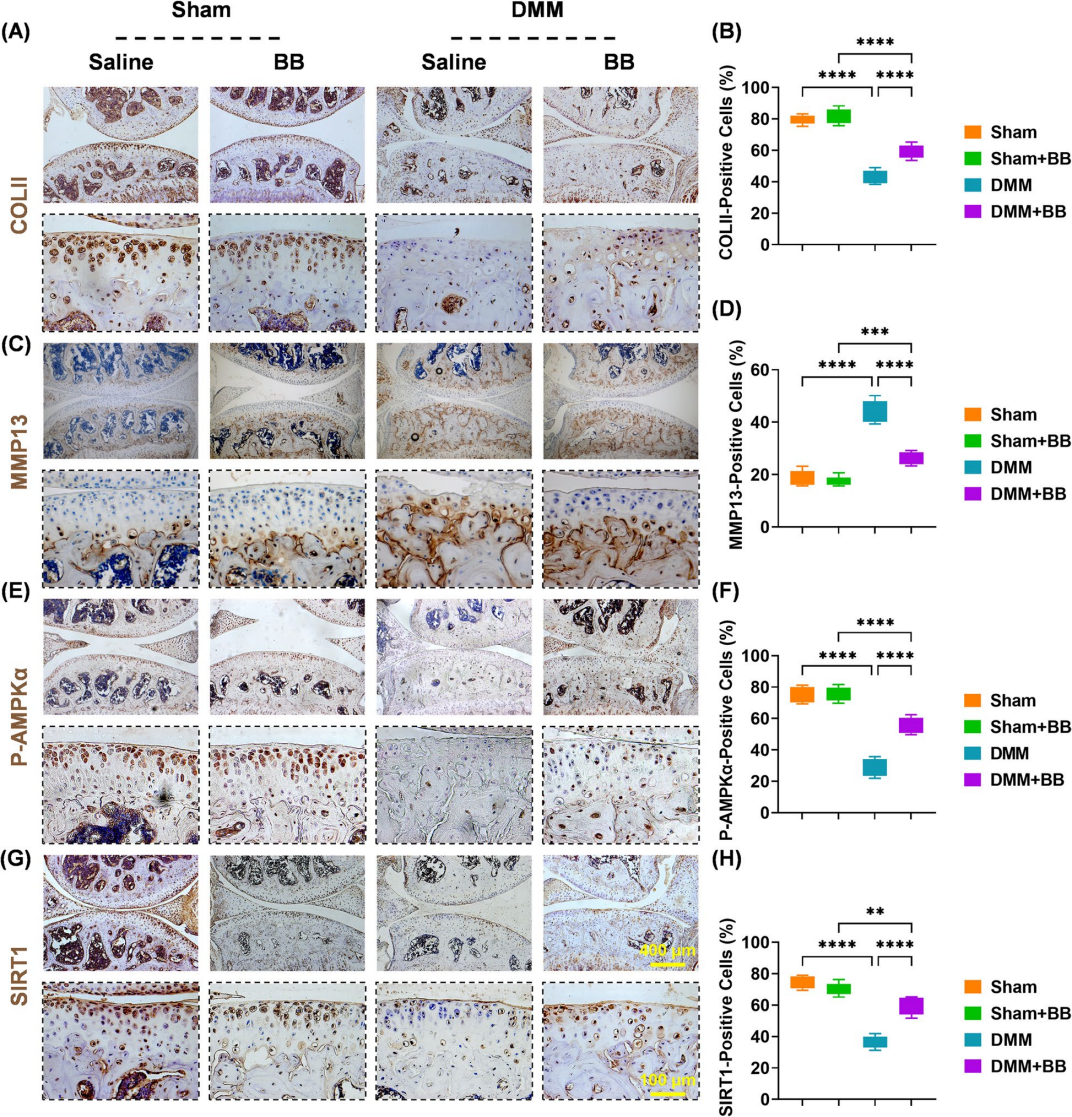
Bilobalide activated AMPK-SIRT1 protected articular cartilage extracellular matrix in vivo. **A**, **B** Representative images of a COLII-stained plot with percentages of COLII-positive cells were quantified. **C**, **D** Representative images of MMP13-stained plot and quantification of the percentage of MMP13-positive cells. **E**, **F** Representative images of phospho (P)-AMPKα-stained plot and quantification of the percentage of P-AMPKα-positive cells. **G**, **H** Representative images of SIRT1-stained plot and quantification of the percentage of SIRT1-positive cells. Each in vivo experiment was repeated six times or more. Statistically, significant differences between the indicated groups are indicated by ^*^p < 0.05, ^**^p < 0.01, ^***^p < 0.001, or ^****^p < 0.0001

We used immunohistochemical (IHC) staining to observe the expression of cartilage ECM markers and AMPK-SIRT1 to investigate the role of the BB-related signaling pathways in vivo. IHC staining indicated that BB treatment increased COLII-positive cells by 36.8% (Fig. 8A, B), but decreased MMP13-positive cells by 39.8% (Fig. 8C, D) to maintain ECM metabolic equilibrium in DMM-induced injured articular cartilage. Additionally, in the DMM + BB group, P-AMPKα and downstream SIRT1 expression levels were improved by 87.7% (Fig. 8E, F) and 64.5% (Fig. 8G, H), respectively, corroborating the activation of AMPK-SIRT1 in vivo after the administration of BB.

## Discussion

Bioinformatics tools that are constantly updated provide new insights for drug development through high-throughput screening, targets prediction, and pathways enrichment. Traditional Chinese herbs have shown to be effective in treating joint disorders such as OA, RA [24], and gouty arthritis [25]; however, the key ingredients responsible for their therapeutic effects and underlying mechanisms by which their effects are manifested are still unknown. According to our bioinformatics results, *Ginkgo biloba* may exert anti-arthritic effects by inhibiting inflammatory cytokines such as IL-6 and modulating oxidative stress proteins such as hypoxia-inducible factor 1 subunit alpha (HIF-1α) and nuclear factor erythroid 2-related factor 2 (NFE2L2, NRF2). *Ginkgo biloba* extract maintains the pro-inflammatory and anti-inflammatory balance by inhibiting hypermethylation of the FOXP3 gene to treat the coal-burning type of arsenism [26]. Similarly, *Ginkgo biloba*-induced anti-inflammatory effects protect hippocampal neurons from trimethyltin-caused acute injury by lowering the expression of TNF-α, IL-1α, IL-6, and inhibiting the nuclear factor kappa B (NF-κB) pathway [27]. Excessive inflammation has been shown to exacerbate cartilage ECM degradation by inhibiting matrix synthesis and activating proteinases activities. *Ginkgo biloba* extract mitigates MMP3 expression levels in IL-1β-stimulated rat chondrocytes [28]; thus, more detailed mechanisms of *Ginkgo biloba-*associated anti-inflammatory effects in OA should be investigated. The development of OA, on the other hand, is dominated by oxidant and antioxidant imbalance, with antioxidant enzymes such as HIF-1α or NRF2 playing a crucial role. Conditional knockout of HIF-1α promotes MMP13 activity and cartilage destruction by interacting with the Wnt/β-catenin pathway [29]. NRF2-knockout mice showed a more severe OA phenotype than wild-type mice, characterized by aggravated cartilage erosion [30], due to impaired antioxidant elements such as heme oxygenase 1 (HO-1) and NAD(P)H quinone dehydrogenase 1 (NQO1). Our latest study demonstrated that the melatonin-activated microRNA (miR)-146a-NRF2-HO-1 axis prevents the progression of OA [4]. Our further research will look into the role of redox status in *Ginkgo biloba*.

In vitro experiments indicated that the lactone BB inspired by *Gingko biloba* had a dual effect on cartilage ECM homeostasis, manifested as increased anabolism and decreased catabolism. Chen et al. reported that 200 μM of BB has a stronger pro-regenerative ability for treating peripheral nerve defects in the area of matrix synthesis [31]. To the best of our knowledge, this study was the first to discover BB restoration on cartilage ECM. The PI3K-AKT pathway is primarily implicated in BB-associated biological effects, according to bioinformatics and RNA sequencing results. Liu et al. revealed that BB inhibits PI3K/AKT signaling, which is followed by NRF2 nuclear translocation and increased translation of downstream antioxidant proteins [32]. Tryosol activates the PI3K/AKT pathway [33], which prevents ECM loss and restores the expression of COLII, ACAN, and SRY-box transcription factor 9 in human nucleus pulposus cells. Considering the importance of PI3K/AKT in cartilage development [34], we speculated that BB-boosted anabolic catabolism may depend on PI3K/AKT, but this needs to be validated. Additionally, BB-induced inhibition of MMPs and ADAMTS should be studied. According to pathway enrichment from RNA sequencing, NF-κB is significantly affected by BB treatment. Zhang et al. demonstrated that BB inhibits the NF-κB signaling pathway, which relieves dextran sulfate sodium-induced colitis [35]. Moreover, BB reduces obesity-related insulin resistance and inflammation by modulating the c-Jun N-terminal kinase (JNK) pathway [36]. Based on this, BB-mediated NF-κB or MAPK inhibition may regulate the activities of ECM proteinases to combat OA. Furthermore, enriched pathways such as ferroptosis, another cell morphological feature involved in the development of OA [37], may play a role in the chondroprotection of BB.

A genomic sequencing technique was used to identify the AMPK-SIRT1 signaling pathway, which was then confirmed by inhibitor experiments. Previous studies have reported that BB-activated AMPK has protective effects against streptozotocin-induced diabetes mellitus injury and lipolysis in 3T3-L1 adipocytes [38]. According to our findings, increased phosphorylation levels of AMPKα proteins may act as a transmitter, amplifying the effects of BB. However, the underlying mechanisms by which BB regulates AMPK are unknown. Overexpression of C1q/tumor necrosis factor-related protects chondrocytes from IL-1β-induced apoptosis by modulating the membrane-located adiponectin receptor (AdipoR1)/AMPK axis [39]. Intriguingly, the adiponectin-AdipoR1-AMPK axis has been linked to MMP3 activation and cartilage destruction [40]. As a longevity code, SIRT1 regulates the chondrogenic fate of mesenchymal stem cells [41] as well as the homeostasis of cartilage ECM synthesis and degradation [42]. The effects of BB on SIRT1 and the underlying mechanisms involved have not been studied. Post-transcriptional or post-translational regulation, such as 3′UTR modification, acetylation, or methylation may be involved; miR-449a-5p [43], miR-34a-5p [44], and miR-30b-5p [45] have been found to be responsible for SIRT1-targeted inhibition. BB regulates the expression of miRNAs such as miR-101-5p and miR-27a-5p, which have multiple biological functions [46]. Synthetically, Both AMPK and SIRT1 are energy-sensing molecules that have coexisted in cells throughout evolution. Upon energy deficit, the AMPK signaling pathway is activated to restore metabolic balance and drive the production of ATP [47]. In turn, SIRT1 is known historically for regulation of longevity due to calorie restriction [48]. Thus, we considered that AMPK/SIRT1 cycle is an interactable loop, of which AMPK-mediated increase in NAD or the NAD/NADH ratio leads to the activation of SIRT1; alternatively, SIRT1 then deacetylates and activates LKB1, which in turn activates AMPK [49]. In this study, inhibition of AMPK with Compound C significantly decreased the expression level and activity of SIRT1, implying the regulatory effects of AMPK on SIRT1 in chondrocytes. According to our previous research, nicotinamide or sirtinol-induced suppression of SIRT1 also negatively affects the phosphorylated level of AMPKα in bone marrow mesenchymal stem cells [50, 51]. Taken together, we inferred that SIRT1 may impact AMPK level in bilobalide-induced anti-arthritic effects; however, additional studies will be performed to further validate this hypothesis.

In addition to reducing cartilage erosion, treatment with BB also reduces subchondral bone sclerosis and the formation of osteophytes. The destruction of cartilage is accelerated by a combination of angiogenesis and osteogenesis in the subchondral bone [52]. Excessive release of angiogenic factors, such as VEGF and platelet-derived growth factor (PDGF)-BB, leads to the formation of type H vessels, angiogenesis, and osteogenesis, all of which contribute to chondrocytes hypertrophy and cartilage destruction [53]. Zhang et al. revealed that BB improves the regeneration of dermal papilla by regulating β-catenin and VEGF expression [54]. Our follow-up study will look into the impact of BB on angiogenesis, especially in type H vessels. Recently, *Ginkgo biloba* extract for treating OA has gained wide attention. Two latest clinical trials (NCT05067998 and NCT05398874) are on recruiting cases to assess the efficacy and safety of the *Ginkgo biloba* extract in patients with knee OA, implying the potential clinical translation of *Ginkgo*-based strategies. Beyond the systemic administration of drugs, local injection such as intra-articular injection is a superior choice for OA treatment. However, injected drugs are slowly absorbed through the peritoneum and transported to articular joints via synovial fluid or blood circulation [55]. A long-term and sustained drug delivery system based on the microfluid platform will be useful for the clinical application of *Ginkgo biloba.* The cumulative release time of candidate drugs can be extended to 4 weeks by assembling them into 100-μm-sized gelatin [56] or hyaluronic acid microspheres [57]; meanwhile, microsphere-provided superlubricity reduces wear and promotes repair. Despite the novel findings, several limitations should be pointed out. First, we mainly focused on one of the bioactive ingredients bilobalide of *Gingko biloba*. In addition, other biomolecules derived from *Gingko biloba* (e.g., diosmetin, genkwanin, and sesamin) may also be capable to antagonize OA but require further investigation. Second, the OA model was established using an instability surgery DMM, other extensively applied procedures such as anterior cruciate ligament transaction (ACLT) or natural aging model will be studied in our future work. Third, as aforementioned, the crosstalk between AMPK and SIRT1 has not been fully determined. Our subsequent experiments will aim to uncover the role of AMPK/SIRT1 cycle in the pathogenesis of OA.

## Conclusions

In this study, we demonstrated that the *Gingko biloba*-inspired lactone BB was found to regulate cartilage ECM metabolic equilibrium by enhancing anabolism and suppressing catabolism. BB treatment in vivo not only reduced cartilage erosion but also mitigated subchondral bone sclerosis. Mechanistically, BB acted as an anti-arthritic agent by upregulating the AMPK-SIRT1 signaling pathway. Our findings led to the discovery of BB, a novel drug that could be used to treat OA.

## Supplementary Information

**Additional file 1: Supplementary Figure 1.** In interleukin (IL)-1β-treated chondrocytes, bilobalide (BB) did not affect cell proliferation. (A-D) Chondrocyte cell viability after treatment with BB or IL-1β. Each in vitro experiment was repeated three times or more. Statistically, significant differences between the indicated groups are indicated by ^*^p < 0.05, ^**^p < 0.01, ^***^p < 0.001, or ^****^p < 0.0001. **Supplementary Figure 2.** Bilobalide modulated extracellular matrix balance in an arthritic environment induced by interleukin-1β. (A-B) Quantitative analysis of COLII or MMP13 fluorescent intensity. (C-F) Quantitative analysis of COLII, ACAN, MMP13, and ADAMTS5 protein levels. Statistically, significant differences between the indicated groups are indicated by ^*^p < 0.05, ^**^p < 0.01, ^***^p < 0.001, or ^****^p < 0.0001. **Supplementary Figure 3.** The AMPK-SIRT1 signaling pathway was improved by bilobalide. (A-B) Quantitative analysis of phospho-AMPK/AMPK and SIRT1 protein levels. Statistically, significant differences between the indicated groups are indicated by ^*^p < 0.05, ^**^p < 0.01, ^***^p < 0.001, or ^****^p < 0.0001. **Supplementary Figure 4.** Compound C inhibits AMPK, thus negating bilobalide-induced cartilage protection. (A-B) Using real-time reverse-transcription-polymerase chain reaction and GAPDH as an internal reference, the transcript levels of *Mmp13* and *Adamts5* were determined. (C-H) Quantitative analysis of phospho-AMPK/AMPK, SIRT1, COLII, ACAN, MMP13, and ADAMTS5 protein levels. Statistically, significant differences between the indicated groups are indicated by ^*^p < 0.05, ^**^p < 0.01, ^***^p < 0.001, or ^****^p < 0.0001.

**Additional file 2: Supplementary Table 1**. Primers used for real-time PCR.

## Data Availability

The data that support the findings of this study are available from the corresponding author upon reasonable request.
