# Supplementary material for: Gingko biloba-inspired lactone prevents osteoarthritis by activating the AMPK-SIRT1 signaling pathway
Source: Arthritis Res Ther. 2022 Aug 18;24:197. doi: 10.1186/s13075-022-02890-y (PMC9387049; doi:10.1186/s13075-022-02890-y)
Supplement: Supplementary file 1 — Additional file 1: Supplementary Figure 1. In interleukin (IL)-1β-treated chondrocytes, bilobalide (BB) did not affect cell proliferation. (A-D) Chondrocyte cell viability after treatment with BB or IL-1β. Each in vitro experiment was repeated three times or more. Statistically, significant differences between the indicated groups are indicated by *p < 0.05, **p < 0.01, ***p < 0.001, or ****p < 0.0001. Supplementary Figure 2. Bilobalide modulated extracellular matrix balance in an arthritic environment induced by interleukin-1β. (A-B) Quantitative analysis of COLII or MMP13 fluorescent intensity. (C-F) Quantitative analysis of COLII, ACAN, MMP13, and ADAMTS5 protein levels. Statistically, significant differences between the indicated groups are indicated by *p < 0.05, **p < 0.01, ***p < 0.001, or ****p < 0.0001. Supplementary Figure 3. The AMPK-SIRT1 signaling pathway was improved by bilobalide. (A-B) Quantitative analysis of phospho-AMPK/AMPK and SIRT1 protein levels. Statistically, significant differences between the indicated groups are indicated by *p < 0.05, **p < 0.01, ***p < 0.001, or ****p < 0.0001. Supplementary Figure 4. Compound C inhibits AMPK, thus negating bilobalide-induced cartilage protection. (A-B) Using real-time reverse-transcription-polymerase chain reaction and GAPDH as an internal reference, the transcript levels of Mmp13 and Adamts5 were determined. (C-H) Quantitative analysis of phospho-AMPK/AMPK, SIRT1, COLII, ACAN, MMP13, and ADAMTS5 protein levels. Statistically, significant differences between the indicated groups are indicated by *p < 0.05, **p < 0.01, ***p < 0.001, or ****p < 0.0001. [file 13075_2022_2890_MOESM1_ESM.docx]

**
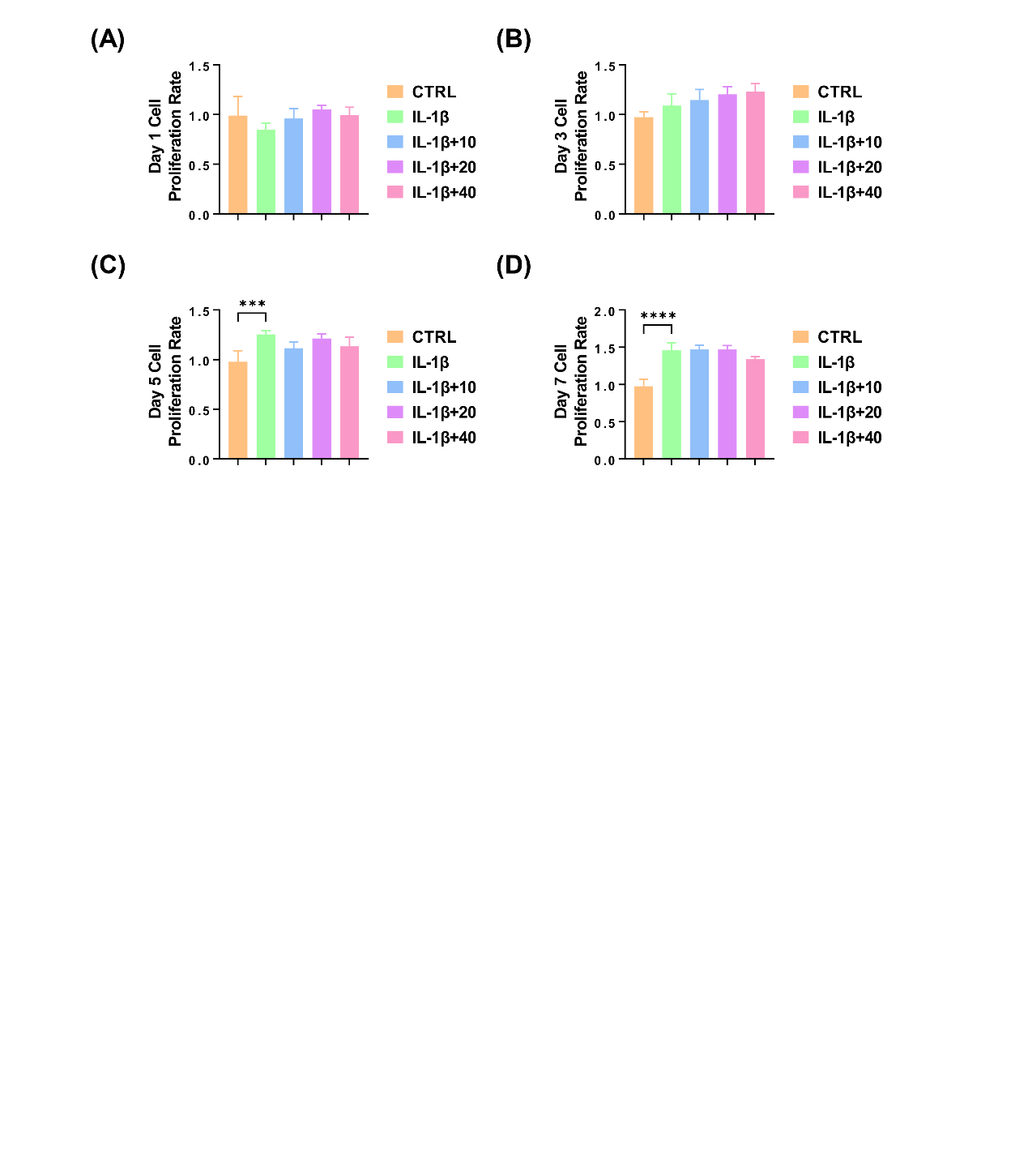
**

**Supplementary Figure 1.** In interleukin (IL)-1β-treated chondrocytes, bilobalide (BB) did not affect cell proliferation. (A-D) Chondrocyte cell viability after treatment with BB or IL-1β. Each *in vitro* experiment was repeated three times or more. Statistically, significant differences between the indicated groups are indicated by ^*^*p* < 0.05, ^**^*p* < 0.01, ^***^*p* < 0.001, or ^****^*p* < 0.0001.


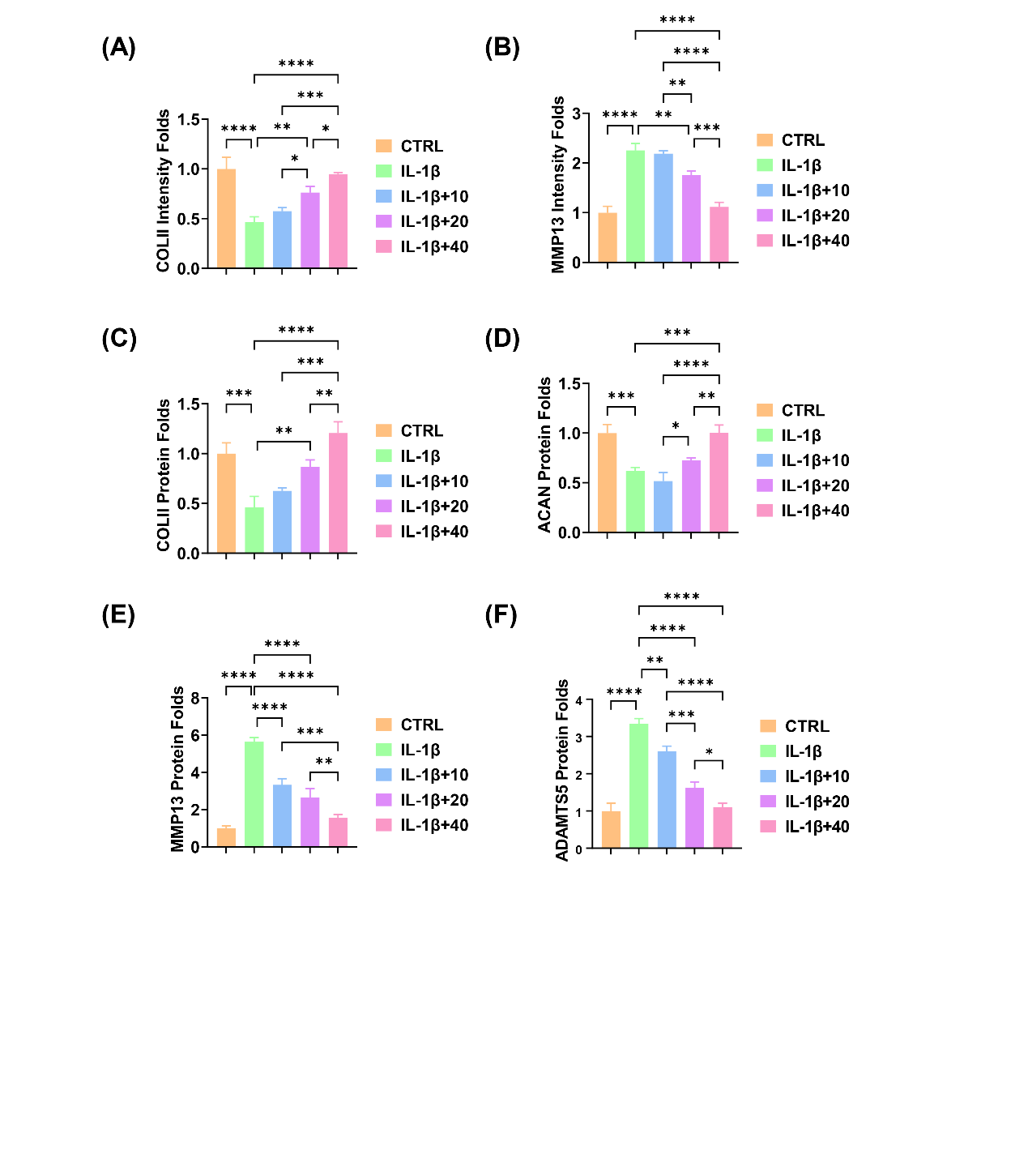


**Supplementary Figure 2.** Bilobalide modulated extracellular matrix balance in an arthritic environment induced by interleukin-1β. (A-B) Quantitative analysis of COLII or MMP13 fluorescent intensity. (C-F) Quantitative analysis of COLII, ACAN, MMP13, and ADAMTS5 protein levels. Statistically, significant differences between the indicated groups are indicated by ^*^*p* < 0.05, ^**^*p* < 0.01, ^***^*p* < 0.001, or ^****^*p* < 0.0001.


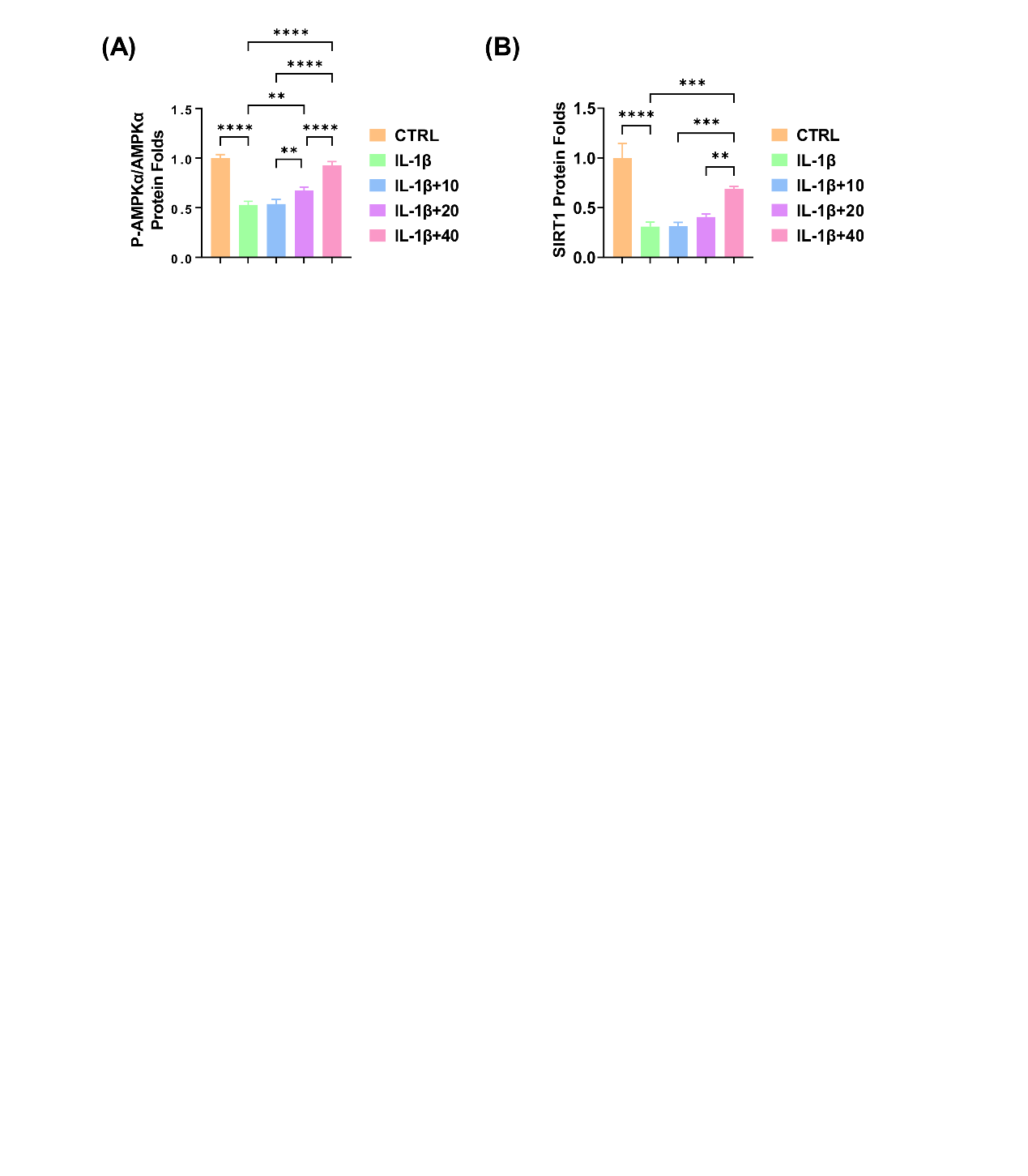


**Supplementary Figure 3.** The AMPK-SIRT1 signaling pathway was improved by bilobalide. (A-B) Quantitative analysis of phospho-AMPK/AMPK and SIRT1 protein levels. Statistically, significant differences between the indicated groups are indicated by ^*^*p* < 0.05, ^**^*p* < 0.01, ^***^*p* < 0.001, or ^****^*p* < 0.0001.


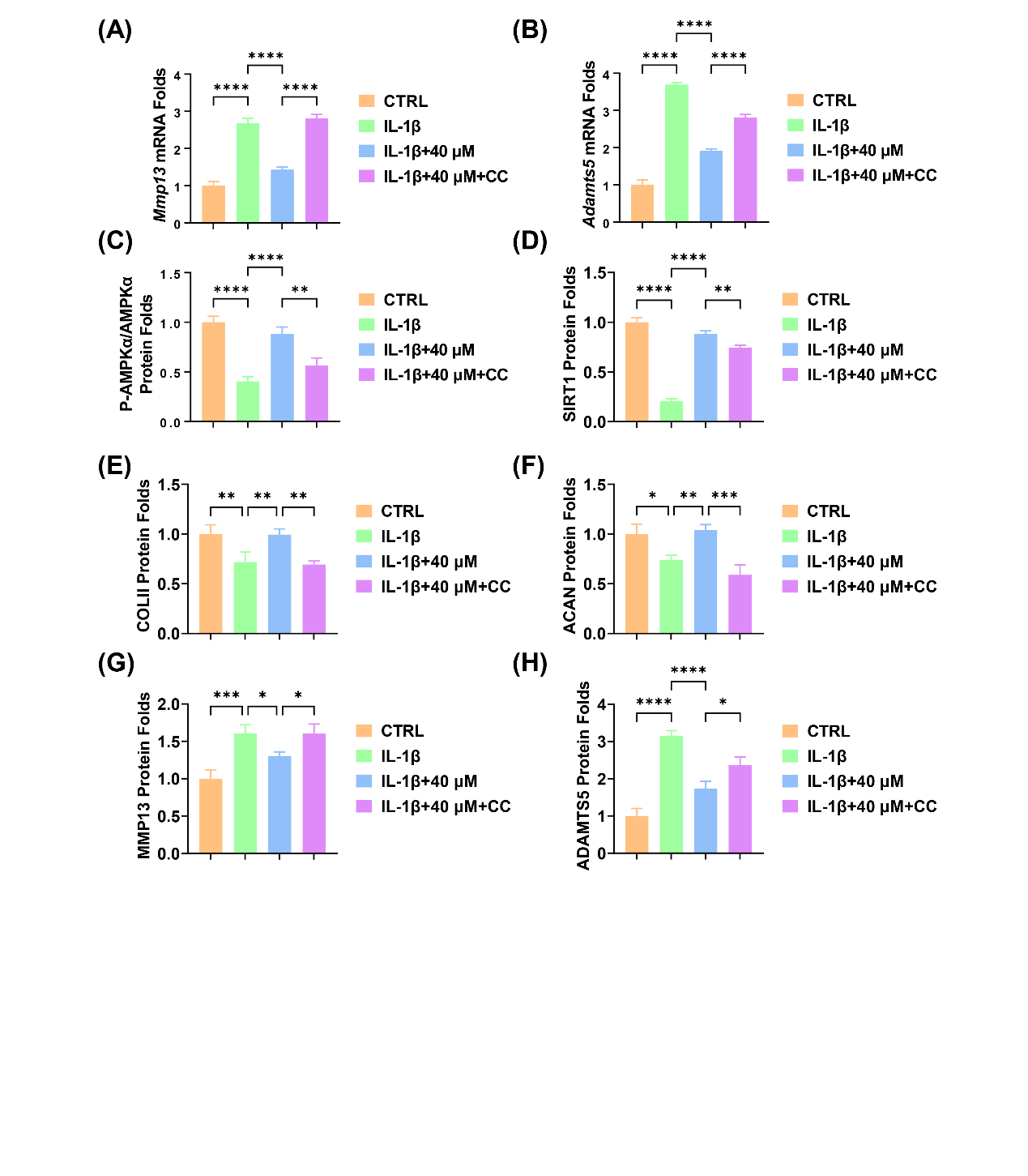


**Supplementary Figure 4.** Compound C inhibits AMPK, thus negating bilobalide-induced cartilage protection. (A-B) Using real-time reverse-transcription-polymerase chain reaction and GAPDH as an internal reference, the transcript levels of *Mmp13* and *Adamts5* were determined. (C-H) Quantitative analysis of phospho-AMPK/AMPK, SIRT1, COLII, ACAN, MMP13, and ADAMTS5 protein levels. Statistically, significant differences between the indicated groups are indicated by ^*^*p* < 0.05, ^**^*p* < 0.01, ^***^*p* < 0.001, or ^****^*p* < 0.0001.
