# Supplementary material for: Gingko biloba-inspired lactone prevents osteoarthritis by activating the AMPK-SIRT1 signaling pathway
Source: Arthritis Res Ther. 2022 Aug 18;24:197. doi: 10.1186/s13075-022-02890-y (PMC9387049; doi:10.1186/s13075-022-02890-y)
Supplement: Supplementary file 2 — Additional file 2: Supplementary Table 1. Primers used for real-time PCR. [file 13075_2022_2890_MOESM2_ESM.docx]

**Supplementary Table 1.** Primers used for real-time PCR

| **Gene** | **Forward Primer sequence (5’-3’)** | **Reverse Primer sequence (5’-3’)** |
| --- | --- | --- |
| *COL2A1* | TGGACGCCATGAAGGTTTTCT | TGGGAGCCAGATTGTCATCTC |
| *ACAN* | ACTCTGGGTTTTCGTGACTCT | ACACTCAGCGAGTTGTCATGG |
| *MMP13* | ACTGAGAGGCTCCGAGAAATG | GAACCCCGCATCTTGGCTT |
| *ADAMTS5*  *SIRT1* | ACTACGATGCAGCTATCCTGT  TAGCCTTGTCAGATAAGGAAGGA | GTCCCAACGTCTGCCATTC  ACAGCTTCACAGTCAACTTTGT |
| *GAPDH* | AGAAAAACCTGCCAAATATGATGAC | TGGGTGTCGCTGTTGAAGTC |
